# Missed opportunities in medical therapy for patients with heart failure in an electronically-identified cohort

**DOI:** 10.1186/s12872-022-02734-2

**Published:** 2022-08-04

**Authors:** Amrita Mukhopadhyay, Harmony R. Reynolds, Arielle R. Nagler, Lawrence M. Phillips, Leora I. Horwitz, Stuart D. Katz, Saul Blecker

**Affiliations:** 1 Leon H. Charney Division of Cardiology, Department of Medicine, New York University Grossman School of Medicine, New York, NY USA; 2 Ronald O. Perelman Department of Dermatology, New York University School Grossman of Medicine, New York, NY USA; 3 Departments of Population Health and Medicine, New York University Grossman School of Medicine, 227 East 30th St., #637, New York, NY 10016 USA

**Keywords:** Heart failure, Guideline-directed medical therapy, Gaps in care, Shortfalls in medical therapy, Electronic cohort

## Abstract

**Background:**

National registries reveal significant gaps in medical therapy for patients with heart failure and reduced ejection fraction (HFrEF), but may not accurately (or fully) characterize the population eligible for therapy.

**Objective:**

We developed an automated, electronic health record-based algorithm to identify HFrEF patients eligible for evidence-based therapy, and extracted treatment data to assess gaps in therapy in a large, diverse health system.

**Methods:**

In this cross-sectional study of all NYU Langone Health outpatients with EF ≤ 40% on echocardiogram and an outpatient visit from 3/1/2019 to 2/29/2020, we assessed prescription of the following therapies: beta-blocker (BB), angiotensin converting enzyme inhibitor (ACE-I)/angiotensin receptor blocker (ARB)/angiotensin receptor neprilysin inhibitor (ARNI), and mineralocorticoid receptor antagonist (MRA). Our algorithm accounted for contraindications such as medication allergy, bradycardia, hypotension, renal dysfunction, and hyperkalemia.

**Results:**

We electronically identified 2732 patients meeting inclusion criteria. Among those eligible for each medication class, 84.8% and 79.7% were appropriately prescribed BB and ACE-I/ARB/ARNI, respectively, while only 23.9% and 22.7% were appropriately prescribed MRA and ARNI, respectively. In adjusted models, younger age, cardiology visit and lower EF were associated with increased prescribing of medications. Private insurance and Medicaid were associated with increased prescribing of ARNI (OR = 1.40, 95% CI = 1.02–2.00; and OR = 1.70, 95% CI = 1.07–2.67).

**Conclusions:**

We observed substantial shortfalls in prescribing of MRA and ARNI therapy to ambulatory HFrEF patients. Subspecialty care setting, and Medicaid insurance were associated with higher rates of ARNI prescribing. Further studies are warranted to prospectively evaluate provider- and policy-level interventions to improve prescribing of these evidence-based therapies.

**Supplementary Information:**

The online version contains supplementary material available at 10.1186/s12872-022-02734-2.

## Introduction

Heart failure is a leading cause for hospitalization in the United States [1], affecting 6.5 million Americans, with substantial morbidity and mortality [2]. Currently, the cornerstone of evidence-based care for patients with heart failure and reduced ejection fraction (HFrEF) includes combination medical therapy that significantly reduces mortality and hospitalization. At least three medication groups are given strong (class I) recommendations for most HFrEF patients by current clinical guidelines [3], are included in published performance measures [4], and supported by large randomized trials [3]. These include (1) beta-blockers (BB); (2) angiotensin converting enzyme-inhibitors (ACE-I), angiotensin receptor blockers (ARB), and angiotensin receptor-neprilysin inhibitors (ARNI); and (3) mineralocorticoid receptor antagonists (MRA).

Under-prescribing of guideline-based medical therapy for HFrEF patients in the United States has previously been described using registry data [5–7]. However, these registries relied on ICD-9 billing codes for patient selection and additional documentation for enrollment, which can result in decreased sensitivity and incomplete capture of potentially eligible patients [8]. Additionally, these studies were conducted before recent guideline updates [3], and therefore, do not reflect updated recommendations for angiotensin receptor-neprilysin inhibitors (ARNI).

We developed an electronic algorithm to identify patients in a large, diverse, urban, multi-site health system with reduced ejection fraction (EF) on echocardiography and without contraindications to guideline-recommended medication classes. The purpose of this study was to generate an electronically-identified cohort using this algorithm, and subsequently assess prescribing patterns of medical therapy and evaluate potential demographic factors associated with under-prescribing of guideline recommended therapy to patients who did not have contraindications.

## Methods

### Study design and setting

We conducted a retrospective cross-sectional study at NYU Langone Health (> 350 ambulatory practice sites). We included patients with EF ≤ 40% on echocardiogram for at least 3 months who were seen by an internal medicine or cardiology provider during the study period 3/1/2019–2/29/2020. We included patients with an echocardiogram performed after 1/1/2017 that had EF reported as a discrete measure. Given that time is needed to add and up-titrate medications, we excluded patients who had a newly diagnosed reduced EF in the last 3 months of the study period. We also excluded patients in whom EF had recovered by the end of the study period, and patients with ventricular assist device, pregnant patients, and those with documented allergy or adverse reaction to the medication class. This study was approved by the NYU Langone Health Institutional Review Board, under a waiver of informed consent.

Because physiologic limitations, such as hypotension, renal dysfunction, and hypokalemia, are noted as possible acceptable reasons to not prescribe therapy [9], we implemented additional exclusion criteria based on medication-specific recommendations [10]. For ACE-I, ARB, ARNI, and MRA, we excluded patients with most recent potassium > 5.1, any potassium > 5.5, and most recent glomerular filtration rate (GFR) < 30 (using the MDRD equation [11] without race modifier [12]). For BB, we excluded patients with most recent heart rate < 60 beats per minute. Because low-normal blood pressure is an acceptable reason to not prescribe medications that lower blood pressure, we excluded patients with systolic blood pressure (SBP) < 105 mmHg at the most recent visit. Current guidelines do not state a specific threshold for SBP when initiating medical therapy [3], and this threshold may vary from patient to patient. Our blood pressure criterion was chosen after consideration by a multidisciplinary team of clinicians as a safe blood pressure threshold at which most patients would tolerate medical therapy and would be acceptable as part of an algorithm to assess clinical practice.

### Electronic identification of patients

Through an iterative process, we developed and refined an electronic algorithm to identify patients with HFrEF who were not on guideline-recommended therapy (Fig. 2A). This algorithm selected patients by using discrete structured fields in our electronic health record (Epic, Epic Systems, Verona, Wisconsin). Echocardiogram reports were generated in Syngo Dynamics (Siemens Medical Solutions, Malvern, Pennsylvania), which includes a structured field for EF; this field is then imported into our electronic health record. Similarly, lab values and blood pressure measurements were also available as discrete structured fields. This allowed us to broadly include all patients that met the inclusion criteria above using the electronic health record alone (Fig. 2A).

We used an iterative process to develop and refine our electronic algorithm. With each iteration of the algorithm, we assessed whether the algorithm accurately identified patients not on guideline-recommended therapy using clinician manual chart review of 20 randomly selected patients for each medication class (total 60 patients per iteration). Any inaccuracies were reviewed with a multidisciplinary team of clinicians, researchers, outpatient administrative leadership, and quality improvement experts, and the algorithm was further refined based on this discussion. This process of manual chart review, multidisciplinary discussion, and algorithm refinement was repeated until no new changes were identified in review, which occurred after three iterations. In the third and final iteration, manual chart review revealed three falsely identified patients: two with improved EF based on data from outside sources and one with an adverse reaction documented in the note, but not in the designated electronic health record field, resulting in a final positive predictive value of 95%.

### Primary outcome

Our primary outcome of interest was whether or not each medication class was prescribed by the end of the study period, based on the eligibility criteria listed above.

### Baseline characteristics and covariates

We collected the following patient demographics: age, gender, race, ethnicity, insurance status, and preferred language. We categorized race as African American (Black), White, Asian, and other, and ethnicity as Hispanic/Latino or Non-Hispanic/Latino based on self-report. We categorized insurance status as Medicare (including managed Medicare), Medicaid (including managed Medicaid), Private (including PPO, EPO, HMO, POS, indemnity, and managed care), and other (including no fault and workers comp). We also collected whether the patient saw a cardiologist in the past year.

### Statistical analysis

Baseline characteristics (age, sex, race, language, insurance status, cardiology visit, and EF) were tabulated for patients eligible for and not eligible for each medication class. The rate of the primary outcome was assessed for the following: (1) BB, (2) ACE-I/ARB/ARNI, (3) ARNI, and (4) MRA. For patients eligible for all medication classes, the number of patients with more than one missing medication class was also assessed. We used standard logistic regression models to assess the effect of the above listed demographic factors and covariates on the odds of prescribing each medication class. All models included the following co-variates: age, sex, race, preferred language (English or not English), insurance status, any cardiology visit during the study period, and EF. Given the high rates of missing data, ethnicity was only included in models to specifically assess the effect of ethnicity on prescribing.

## Results

### Patient characteristics

**Fig. 1:**
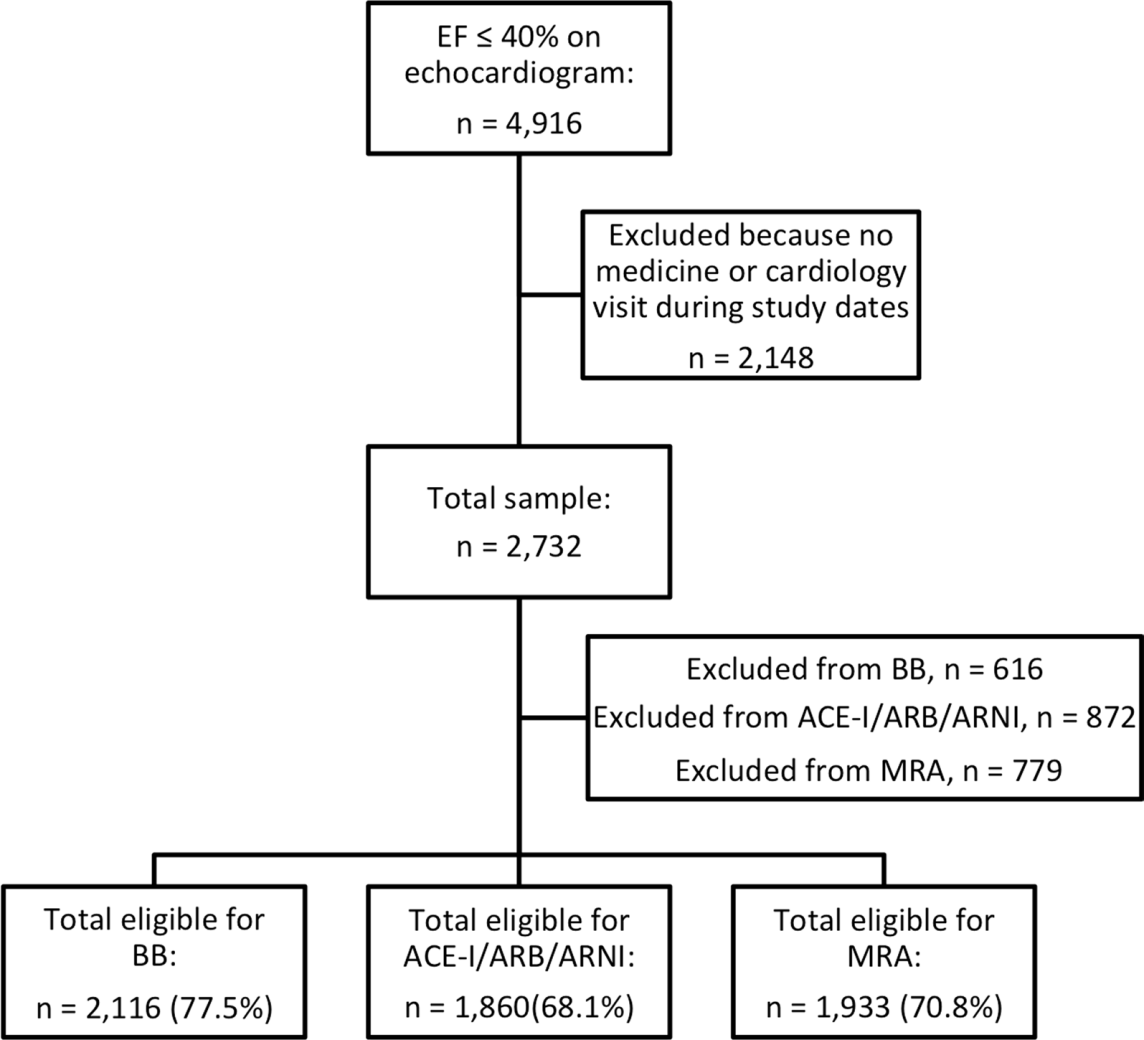
Patient flow diagram

**Fig. 2:**
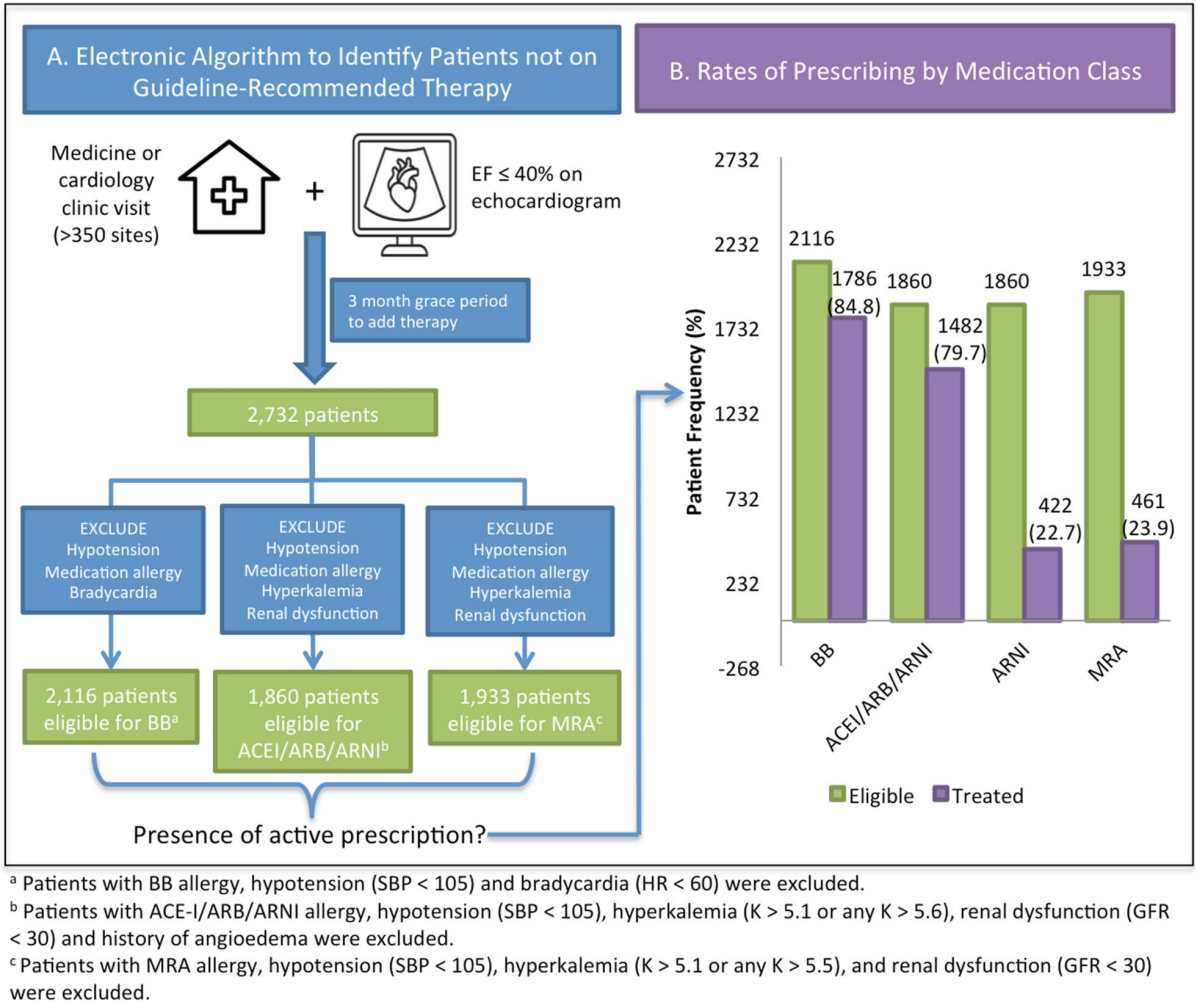
Electronic algorithm to identify heart patients eligible for, but not prescribed, appropriate medical therapy (**A**), and associated rates of prescribing by medication class (**B**)

A total of 2732 patients with EF ≤ 40% and an eligible visit during the study period were electronically identified for inclusion in the study. Of these patients, 77.5% (n = 2116) of the patients were eligible for BB therapy, 68.1% (n = 1860) for ACE-I/ARB/ARNI therapy, and 70.8% (n = 1933) for MRA therapy (Figs. 1 and 2A). Table 1 summarizes baseline characteristics for patients eligible for each medication class and Additional files 1, 2, Table 1 includes baseline characteristics of patients who were or were not eligible for each medication class. Overall, the average age was 70.0 years, and the majority of patients were male sex (71.2%), White race (70.6%), English-speaking (81.4%), and had Medicare insurance (65.6%). Almost all patients (94.6%) had seen a cardiologist in the past year.

**Table 1:** Baseline characteristics

Mean ± std. dev. or % (N)	Total sample (n = 2732)	Eligible for BB (n = 2116)	Eligible for ACE/ARB/ARNI (n = 1860)	Eligible for MRA (n = 1933)
Age (years)	70.0 ± 13.7	69.8 ± 13.7	69.9 ± 13.6	69.8 ± 13.6
Sex—% male (n)	71.2% (1945)	70.6% (1494)	71.4% (1328)	71.3% (1737)
Race	n = 2604	n = 2018	n = 1769	n = 1838
White	70.6% (1837)	70.0% (1413)	72.1% (1276)	72.1% (1326)
Black	12.8% (333)	13.4% (271)	12.2% (216)	12.5% (229)
Asian	4.8% (125)	4.4% (89)	3.9% (69)	3.9% (72)
Other	11.9% (309)	12.1% (245)	11.8% (208)	11.5% (211)
Ethnicity	n = 427	n = 324	n = 266	n = 277
Non-Hispanic	91.6% (391)	91.9% (298)	92.1% (245)	92.1% (255)
Hispanic	8.4% (36)	8.0% (26)	7.9% (21)	7.9% (22)
Language	n = 2721	n = 2107	n = 1851	n = 1924
English	81.4% (2215)	81.1% (1709)	80.8% (1496)	81.3% (1564)
Other	18.6% (506)	18.9% (398)	19.2% (355)	18.7% (360)
Insurance	n = 2700	n = 2091	n = 1837	n = 1910
Medicare	65.6% (1772)	64.6% (1351)	64.0% (1175)	64.1% (1224)
Private	24.9% (672)	25.2% (526)	26.4% (484)	26.4% (505)
Medicaid	9.3% (252)	10.0% (210)	9.5% (174)	9.3% (177)
Other	0.2% (4)	0.2% (4)	0.2% (4)	0.2% (4)
Cardiology visit in past year	94.6% (2584)	94.2% (1994)	94.8% (1764)	94.8% (1833)
Ejection fraction (%)	32.6 ± 7.3	32.9 ± 7.1	33.1 ± 6.9	33.1 ± 7.0

### Rates of Prescribing of Medical Therapy

Figure 2B depicts rates of prescribing by medication class. Among 2,116 patients eligible for BB (77.5% of overall cohort), 84.8% were prescribed a BB. Among 1,860 patients eligible for ACE-I, ARB or ARNI (68.1% of the cohort), 79.7% were prescribed one of these agents. However, ARNI was used in just 22.7% of these cases. Additionally, among 1,933 patients eligible for MRA (70.8% of the cohort), 23.9% were prescribed MRA.

In a subgroup analysis of 1684 patients (61.6% of overall cohort) who were eligible for all three medication groups (BB, ACEI/ARB/ARNI, MRA), 349 patients (20.7%) were prescribed agents from all three groups, of whom 171 patients (49.0%) were prescribed ARNI. When at least one medication group was not prescribed (n = 1335), it was nearly always the case that MRA was not prescribed (1283 patients, 96.1%). Similarly, among the 484 patients (27.8%) lacking either ACE-I/ARB/ARNI or BB prescription, the majority (432 patients, 89.3%) were also lacking an MRA prescription.

### Factors associated with medication prescribing (Table 2)

**Table 2:** Adjusted odds of prescribing medical therapy

	BB (n = 1991)^b^	ACE-I/ARB/ARNI (n = 1740)^a^	ARNI (n = 1744)^c^	MRA (n = 1809)^d^
Adjusted^e^ odds of prescribing therapy, OR (95% CI)				
Age (years)	0.99 (0.98–1.0)	0.99 (0.97–0.99)*	0.99 (0.98–1.0)*	0.98 (0.97–0.99)**
Sex	–	–	–	–
Male	1.04 (0.79–1.37)	0.86 (0.66–1.12)	0.97 (0.72–1.2)	0.78 (0.61–1.0)
Race				
White	–	–	–	**–**
Black	1.03 (0.59–1.53)	1.40 (0.91–2.16)	1.3 (0.85–1.7)	1.5 (1.0–2.0)*
Other	0.96 (0.66–1.4)	1.34 (0.93–1.94)	1.0 (0.75–1.5)	1.6 (1.2–2.2)**
Ethnicity^d^				
Non-Hispanic	–	–	–	–
Hispanic	0.57 (0.17–1.9)	1.06 (0.35–3.2)	0.41 (0.08–2.04)	0.28 (0.06–1.30)
Language				
English	**–**	–	–	**–**
Other	1.6 (1.1–2.3)*	1.3 (0.96–1.83)	0.95 (0.69–1.3)	0.61 (0.43–0.85)**
Insurance				
Medicare	–	**–**	**–**	–
Private	1.06 (0.62–1.80)	1.50 (1.04–2.17)*	1.4 (1.02–2.00)*	1.1 (0.78–1.5)
Medicaid	0.93 (0.64–1.33)	0.86 (0.52–1.42)	1.70 (1.07–2.67)*	1.1 (0.69–1.7)
Cardiology visit	2.23 (1.43–3.47)***	2.62 (1.65–4.17)***	5.9 (2.3–14.8)***	6.09 (2.4–15.3)***
EF (%)	0.97 (0.96–0.99)*	0.98 (0.95–0.99)*	0.93 (0.92–0.95)***	0.93 (0.91–0.95)***

Bold indicates statistical significance

*p < 0.05

**p < 0.005

***p < 0.001

^a^120 not included because of missing co-variate data

^b^125 not included because of missing co-variate data

^c^116 not included because of missing co-variate data

^d^124 not included because of missing co-variate data

^e^All models adjusted for age, sex, race, language, insurance status, cardiology visit, and EF (ejection fraction). Given the high rates of missing data, ethnicity was only included in models to specifically assess the effect of ethnicity on prescribing. Models including ethnicity had sample size of 258, 311, 261, and 262 for ACE-I/ARB/ARNI, BB, ARNI, and MRA respectively

Patients were less likely to be prescribed ACE-I/ARB/ARNI or MRA with increasing age (OR = 0.99 per year, 95% CI = 0.97–0.99, p < 0.05; and OR = 0.98, 95% CI = 0.97–0.99, p < 0.005, respectively). Compared to White patients, Black patients and patients with other race were more likely to be prescribed MRA (OR = 1.5, 95% CI = 1.0–2.0, p < 0.05, and OR = 1.6, 95% CI = 1.2–2.2, p < 0.005, respectively). Compared to patients who spoke English, non-English-speaking patients were less likely to be prescribed an MRA (OR = 0.61, 95% CI = 0.43–0.85, p < 0.005), but more likely to be prescribed BB (OR = 1.6, 95% CI = 1.1–2.3, p < 0.005). Additionally, compared to patients with Medicare, patients with private insurance and Medicaid were more likely to be prescribed ARNI (OR = 1.4, 95% CI = 1.02–2.00, p < 0.05; and OR = 1.70, 95% CI = 1.07–2.67, p < 0.05, respectively). Cardiology visit and lower EF were associated with increased prescribing of all medication classes.

## Discussion

In our retrospective, cross-sectional study of 2732 electronically-identified outpatients with HFrEF in a large, diverse, health system with over 350 ambulatory practices, less than a quarter of eligible patients were prescribed MRA and ARNI. This was observed despite the fact that our study was conducted after the HFrEF guidelines updates in 2017 and the subsequently increased awareness of newer medical therapies. Younger age, lower EF, and cardiology visit were associated with increased prescribing of most medication classes. Surprisingly, Black race was associated with more frequent MRA prescribing, and Medicaid insurance was associated with greater ARNI prescribing.

Our study identified patients using an electronic algorithm based on discretely coded EF on echocardiography, thereby capturing a broad clinical population. Our electronic algorithm accounted for and excluded patients with potential physiologic barriers to prescribing (i.e., bradycardia, hypotension, renal dysfunction, hyperkalemia). This selection method differed from other registries [5–7, 13–16], many of which did not account for physiologic barriers, and used ICD billing and diagnosis codes to select patients, a method that has been shown to incompletely capture patients with HFrEF [8]. Some registries also had added documentation and follow up requirements as well as specific inclusion criteria, which further limited their ability to capture a broad clinical population. Despite these differences, our cohort had similar rates of prescribing (BB: 84.8%, ACE-I/ARB/ARNI: 79.7%, ARNI: 22.7%, MRA 23.9%) as compared to prior HFrEF registries in the United States (BB: 67–86%, ACE-I/ARB/ARNI: 60.5–80.0%, ARNI: 13.0%, MRA: 33.4–36%) [5, 7], and lower rates of MRA prescribing as compared to prior international registries (BB: 79–86.7%, ACE-I/ARB/ARNI: 77–88.5%, MRA: 43.7–69.3%) [13–16]. While some have noted physiologic limitations as a possible reason for these treatment gaps [9], our algorithm accounts for such limitations as hypotension, bradycardia, hyperkalemia, and renal dysfunction. Unfortunately, despite accounting for physiologic limitations, we still observed substantial shortfalls in therapy. Additionally, our findings, obtained from a broad, contemporary clinical population indicates an imperative need for widespread study of barriers to and implementation of interventions to improve prescribing of these life-saving therapies.

Financial barriers and insurance coverage are often proposed as reasons for non-prescription [17]. Our finding of differential rates of prescribing by insurance status supports this claim. We found that patients with Medicaid insurance were more likely to be prescribed ARNI than those with Medicare. This could be reflective of differences in prior authorization requirements between the two types of plans, specifically in New York. Prior authorization for ARNI is not required for HFrEF patients with New York State Medicaid [18], but can be required for over a third of Medicare plans [19]. Our results suggest that policy decisions around prior authorization, coverage, and co-payments could have an impact on the prescribing of guideline-recommended, life-saving therapies at a population level. This finding supports changing prior authorization requirements to improve the use of guideline-recommended therapies.

In addition to potential insurance-related barriers, we examined racial inequities in prescribing. Our finding of higher rates of MRA prescribing for Black patients with HFrEF stands in contrast to prior studies demonstrating under-prescription of guideline-recommended therapies to Black patients as compared to White patients in other disease states, such as anticoagulation for atrial fibrillation [20–22] and lipid-lowering therapy for primary prevention [23–25]. A prior U.S. registry study also found higher prescription rates for MRA in Black patients compared to White patients with HFrEF [5]. One possible reason for this consistent finding may be the higher incidence of resistant hypertension amongst Black patients [26], given that MRA can be an effective additional agent [27]. Another explanation may be the complex relationship between race, creatinine, and calculated GFR. The MDRD equation for GFR has traditionally included a multiplier for Black race, which if used, results in a higher estimation of a patient’s GFR, and therefore, less sensitive detection of renal dysfunction in Black patients [12]. While we did not use the race multiplier in our study for this reason, prescribing physicians had the ability to view both calculations of GFR in the electronic health record, and may have been more comfortable prescribing MRA in patients with borderline renal function if using the higher, race-adjusted, GFR estimation.

In order to explore the potential for targeted interventions, we also assessed whether a cardiology visit was associated with higher rates of prescribing, and examined the overlap between prescribing of the different medication classes. While cardiology visit was strongly associated with improved prescribing of all medication classes, consistent with prior studies [28], over 90% of patients had seen a cardiologist, suggesting that universal referral to cardiologist is insufficient to achieve guideline concurrent care. Additionally, we found that majority of patients who were not appropriately prescribed BB or ACE-I/ARB, were also not prescribed MRA, indicating that interventions targeted to providers with low rates of MRA prescribing could also be tailored to improve prescribing of the other therapies as well. Notably, among the medical therapies studied here, MRA has the lowest number needed to treat for mortality based on randomized data [29], further supporting interventions to target providers to improve prescribing of this medication class.

Overall, while our findings of persistent gaps in medical therapy as compared to historical registry data could be interpreted as discouraging, further improvement is both achievable and necessary. Of note, a recent study conducted at a single HF specialty clinic in Canada found higher rates of prescribing of HFrEF medical therapy compared to previously published registries (BB: 98.6%, ACE-I/ARB/ARNI: 82.9%, ARNI: 91.4%, MRA: 93.4%), despite more stringent study criteria [9]. This clinic utilized multiple evidence-based techniques such as close follow up and multi-disciplinary leadership with heart failure subspecialists and pharmacists [30–32]. While this type of model may be costly, our data supports a need to implement proven interventions to improve outcomes for these patients. Concurrently, alternative, lower cost interventions should continue to be investigated. These may include electronic health record-based interventions [33] and patient engagement tools [34].

### Study limitations

Our results should be interpreted in the context of several limitations. Our study was a cross-sectional, retrospective analysis, and was therefore limited in its ability to draw causal conclusions. Additionally, while our sample consisted of hundreds of different ambulatory practices, ranging from academic hospitals to community clinics, data was obtained from a single health system, and may therefore be limited in generalizability. In addition, our models did not include potential clinical confounders, such as BMI, hypertension, and hospitalization. We also did not assess medication doses, an important aspect of optimizing medical therapy in HFrEF. We excluded patients with SBP < 105 mmHg, which could limit generalizability of our findings to patients with lower blood pressure, some of whom may be able to tolerate medical therapy. Finally, although our electronic algorithm allowed us to capture a broad patient population, it limited our ability to obtain more granular data not reliably documented in the electronic record, such as functional status or etiology of heart failure, which may effect prescribing decisions.

## Conclusions

In our diverse, urban, electronically-identified patient population with HFrEF, most of whom were seen by a cardiologist in the preceding year, gaps in medication prescribing were most notable for MRA and ARNI. Under-prescribing of MRA was independently associated with older age, White race, lower EF, lack of a cardiology visit, and preferred language other than English and under-prescribing of ARNI was independently associated with older age, lower EF, lack of a cardiology visit, and Medicare insurance. Our findings highlight the need for systems-level quality improvement efforts to reduce these gaps in care. Such efforts could include elimination of requirement for prior authorization for ARNI, as demonstrated by greater prescribing of ARNI to patients with New York State Medicaid as compared to Medicare in our study. Our results indicate an urgent need for study and implementation of comprehensive interventions to improve MRA and ARNI prescribing for patients with HFrEF.

## Supplementary Information

**Additional file 1.** Baseline Characteristics for Eligible and Ineligible Patients.

**Additional file 2.** Acceptable Medications.

## Data Availability

The datasets generated and/or analysed during the current study are not publicly available due as they include protected health information, but may be available from the corresponding author on reasonable request with IRB approval.

## Abbreviations

ACE-I: Angiotensin converting enzyme-inhibitor
ARB: Angiotensin receptor blocker
ARNI: Angiotensin receptor-neprilysin inhibitor
BB: Beta blocker
EF: Ejection fraction
GFR: Glomerular filtration rate
HFrEF: Heart failure with reduced ejection fraction
