# Supplementary material for: Missed opportunities in medical therapy for patients with heart failure in an electronically-identified cohort
Source: BMC Cardiovasc Disord. 2022 Aug 4;22:354. doi: 10.1186/s12872-022-02734-2 (PMC9354331; doi:10.1186/s12872-022-02734-2)
Supplement: Supplementary file 1 — Additional file 1. Baseline Characteristics for Eligible and Ineligible Patients. [file 12872_2022_2734_MOESM1_ESM.docx]

**Additional File 1.** Baseline Characteristics for Eligible and Ineligible Patients

| Mean ± std. dev. or % (N) | Total sample  (n=2,732) | Eligible for ACE/ARB/ARNI (n=1,860) | Ineligible for ACE/ARB/ARNI (n=872) | Eligible for BB (n=2,116) | Ineligible for BB (n=616) | Eligible for MRA (n=1,933) | Ineligible for MRA (n=799) |
| --- | --- | --- | --- | --- | --- | --- | --- |
| **Age (years)** | 70.0 ± 13.7 | 69.9 ± 13.6 | 70.3 ± 14.1 | 69.8 ± 13.7 | 70.6 ± 13.8 | 69.8 ± 13.6 | 70.5 ± 14.2 |
| **Sex – % male** | 71.2% (1,945) | 71.4% (1,328) | 70.8% (617) | 70.6% (1,494) | 73.2% (451) | 71.3% (1,737) | 71.6% (572) |
| **Race**   - **White** - **Black** - **Asian** - **Other** | n=2,604  70.6% (1,837)  12.8% (333)  4.8% (125)  11.9% (309) | n=1,769  72.1% (1,276)  12.2% (216)  3.9% (69)  11.8% (208) | n=835  67.1% (561)  14.0% (117)  6.7% (56)  12.1% (101) | n=2,018  70.0% (1,413)  13.4% (271)  4.4% (89)  12.1% (245) | n=586  72.4% (424)  10.6% (62)  6.1% (36)  10.9% (64) | n=1,838  72.1% (1,326)  12.5% (229)  3.9% (72)  11.5% (211) | n=766  66.7% (511)  13.6% (104)  6.9% (53)  12.8% (98) |
| **Ethnicity**   - **Non-Hispanic** - **Hispanic** | n=427  91.6% (391)  8.4% (36) | n=266  92.1% (245)  7.9% (21) | n=161  90.7% (146)  9.3% (15) | n=324  91.9% (298)  8.0% (26) | n=103  90.3% (93)  9.7% (10) | n=277  92.1% (255)  7.9% (22) | n=150  90.7% (136)  9.3% (14) |
| **Language**   - **English** - **Other** | n=2,721  81.4% (2,215)  18.6% (506) | n=1,851  80.8% (1,496)  19.2% (355) | n=870  82.6% (719)  17.4% (151) | n=2,107  81.1% (1,709)  18.9% (398) | n=614  82.4% (506)  17.6% (108) | n=1,924  81.3% (1,564)  18.7% (360) | n=797  81.7% (651)  18.3% (146) |
| **Insurance**   - **Medicare** - **Private** - **Medicaid** - **Other** | n=2,700  65.6% (1,772)  24.9% (672)  9.3% (252)  0.2% (4) | n=1,837  64.0% (1,175)  26.4% (484)  9.5% (174)  0.2% (4) | n=863  69.2% (597)  21.8% (188)  9.0% (78)  0.0% (0) | n=2,091  64.6% (1,351)  25.2% (526)  10.0% (210)  0.2% (4) | n=609  69.1% (421)  24.0% (146)  6.9% (42)  0.0% (0) | n=1,910  64.1% (1,224)  26.4% (505)  9.3% (177)  0.2% (4) | n=790  69.4% (548)  21.1% (167)  9.5% (75)  0.0% (0) |
| **Cardiology visit in past year** | 94.6% (2,584) | 94.8% (1,764) | 94.0% (820) | 94.2% (1,994) | 95.8% (590) | 94.8% (1,833) | 93.4% (751) |
| **Ejection fraction (%)** | 32.6 ± 7.3 | 33.1 ± 6.9 | 31.4 ± 7.8 | 32.9 ± 7.1 | 31.4 ± 7.7 | 33.1 ± 7.0 | 31.3 ± 7.9 |
