# Supplementary material for: Missed opportunities in medical therapy for patients with heart failure in an electronically-identified cohort
Source: BMC Cardiovasc Disord. 2022 Aug 4;22:354. doi: 10.1186/s12872-022-02734-2 (PMC9354331; doi:10.1186/s12872-022-02734-2)
Supplement: Supplementary file 2 — Additional file 2. Acceptable Medications. [file 12872_2022_2734_MOESM2_ESM.docx]

**Additional File 2**

**Acceptable Medications**

The following medications were considered acceptable for each medication class (note, long-acting, short-acting, brand-name, and combination-pill formulations were all included):

1. ACE-I – benazepril, captopril, enalapril, enalaprilat, fosinopril, lisinopril, moexipril, perindopril, quinapril, ramipril, trandolapril
2. ARB – azilsartan, candesartan, eprosartan, irbesartan, losartan, olmesartan, telmisartan, valsartan
3. ARNI – sacubritil/valsartan
4. BB – bisoprolol, carvedilol, metoprolol (succinate or tartrate)
5. MRA – epleronone, spironolactone
